# Exploring Academic Performance of Medical Students in an Integrated Hybrid Curriculum by Gender

**DOI:** 10.1007/s40670-023-01743-w

**Published:** 2023-02-07

**Authors:** DeLoris Wenzel Hesse, Lynn M. Ramsey, Lia Pierson Bruner, Claudia S. Vega-Castillo, Dina Teshager, Janette R. Hill, Mary T. Bond, Edwin V. Sperr, Amy Baldwin, Amy E. Medlock

**Affiliations:** 1 Augusta University/University of Georgia Medical Partnership, Athens, GA USA; 2 Department of Cellular Biology, University of Georgia, Athens, GA USA; 3 Department of Family and Community Medicine, Medical College of Georgia, Augusta University, Augusta, GA USA; 4 College of Education, Learning, Design, & Technology, University of Georgia, Athens, GA USA; 5 Department of Medicine: General Internal Medicine, Medical College of Georgia, Augusta University, Augusta, GA USA; 6 Department of Biochemistry and Molecular Biology, Medical College of Georgia, Augusta University, Augusta, GA USA; 7 Department of Biochemistry and Molecular Biology, University of Georgia, Athens, GA USA

**Keywords:** Evaluation, Gender gap, Integrated, Medicine, Summative assessment, Undergraduate

## Abstract

Gender gaps in academic performance have been reported at a variety of educational levels including several national standardized exams for medical education, with men scoring higher than women. These gaps potentially impact medical school acceptance and residency matching and may be influenced by curricular design. Performance data for our 4-year integrated hybrid curriculum, which features a large proportion of active learning, revealed a gender gap with men performing better early in the curriculum and on the first national standardized exam. This gap in performance almost entirely disappeared for years 2–4 of the curriculum and the second national standardized exam.

## Background

Women continue to be underrepresented in science, technology, engineering, and mathematics (STEM) fields, notably in leadership positions [1, 2]. Gender achievement gaps in education generate differences in the accumulation of human capital and available career opportunities [3]. Educational gender gaps have been noted at a variety of levels including medical education, where men score higher than women on the Medical College Admission Test (MCAT) (science portion) [4] and the United States Medical Licensing Examination (USMLE) Step 1 [5]. Typically, this gender gap closes or reverses slightly by the USMLE Step 2 Clinical Knowledge (CK) [6]. Understanding the gender gap in academic performance is crucial considering the major role these exams have played in candidate selection for medical school admission and residency training.

The Augusta University/University of Georgia Medical Partnership, a 4-year campus of the Medical College of Georgia, opened in 2010. Our pre-clerkship curriculum is a hybrid of many learning formats including large groups, case-based small groups, anatomy laboratories, simulation, clinical skills, community and population health via service learning and community-based group projects, and self-directed learning opportunities [7–9]. Our first-year students complete five integrated system–based modules, and the second-year students complete six. Clinical experience starts in the first year at community sites and continues in years 3 and 4 with typical clerkships and electives.

A gender gap in academic performance has been reported in undergraduate science courses, associated with multiple factors including class size, assessment type, and pedagogy, with women scoring lower than men [10, 11]. Additionally, the gender gap in performance has been shown to exist on multiple levels in undergraduate courses that use active learning [12, 13]. Since a majority of our pre-clerkship, hybrid curriculum utilizes active learning strategies, we designed this study to determine if a gender gap in performance exists at any point in the training at our institution.

## Activity

We compared 8 years of data from our students’ internal and national summative exams from classes that matriculated in 2011 to 2019 (*n* = 358 students, 40% women overall) (Table 1). Data included pre-matriculation factors (overall grade point average (GPA), science GPA, and MCAT scores), pre-clerkship summative assessments (board-style multiple choice questions in years 1 and 2), USMLE Step 1 and Step 2 CK, and National Board of Medical Examiners (NBME) subject exams reported as equated percent correct score (years 3 and 4). Internal summative assessments included module and semester final exams, objective structured clinical examinations (OSCEs), and anatomy final exams. Data were collated, sorted by gender, and de-identified. Individuals with incomplete data for year 1 or 2 were removed prior to analysis. Mean, standard deviation, and count were determined for men and women. Data were analyzed using an independent sample *t* test and two-way factor ANOVA. Statistical significance was defined as *p* < 0.05. The study received approval from the UGA Institutional Review Board (PROJECT00000557).

**Table 1:** Summary of matriculating class size and composition by year

**Matriculation year**	**Men**	**Women (%)**
**2011**	27	12 (31)
**2012**	28	13 (32)
**2013**	24	14 (37)
**2014**	25	14 (36)
**2015**	22	18 (45)
**2016**	24	16 (40)
**2017**	21	19 (48)
**2018**	21	20 (49)
**2019**	22	18 (45)
**Total**	214	144 (40)

## Results and Discussion

To determine if there were differences in pre-matriculation data, we analyzed overall GPA, science GPA, and MCAT scores from students accepted to our campus who began their training from 2011 to 2019. When sorted by gender, these data demonstrated that there was a statistically significant difference in the science GPA and in MCAT scores (new format) between men and women, with men scoring higher than women. However, there was not a significant difference in overall GPA or MCAT scores (old format) (Table 2).

**Table 2:** Assessment items analyzed

**Assessment**	**Group**	**Mean ± SD**	***pvalue***	**95% CI**
**MCAT Old**	Men: *N* = 69	31.2 ± 3.3	0.0533	
	Women: *N* = 48	29.9 ± 3.6		
**MCAT New**	Men: *N* = 58	512 ± 4.8	0.0034	1.012 to 4.988
	Women: *N* = 54	509 ± 5.8		
**Overall GPA**	Men: *N* = 133	3.74 ± 0.20	0.0758	
	Women: *N* = 103	3.70 ± 0.23		
**Science GPA**	Men: *N* = 87	3.69 ± 0.25	0.0429	0.0030 to 0.1770
	Women: *N* = 69	3.60 ± 0.30		
**Year 1 Module 1**	Men: *N* = 133	85.5 ± 6.3	0.0105	0.473 to 3.527
	Women: *N* = 102	83.5 ± 5.3		
**Year 1 Module 1 Anatomy**	Men: *N* = 133	69.4 ± 15	0.4350	
	Women: *N* = 102	67.9 ± 14		
**Year 1 Module 2**	Men: *N* = 133	84.4 ± 6.3	0.0501	
	Women: *N* = 102	82.8 ± 6.0		
**Year 1 Module 2 Anatomy Part 1**	Men: *N* = 133	75.2 ± 13	0.0164	0.739 to 7.261
	Women: *N* = 102	71.2 ± 12		
**Year 1 Module 2 Anatomy Part 2**	Men: *N* = 133	77.9 ± 11	0.0985	
	Women: *N* = 102	75.3 ± 13		
**Year 1 Module 3**	Men: *N* = 133	81.9 ± 7.1	0.0303	0.192 to 3.808
	Women: *N* = 102	79.9 ± 6.8		
**Year 1 Module 3 Anatomy**	Men: *N* = 133	69.1 ± 16	0.8455	
	Women: *N* = 102	68.7 ± 15		
**Year 1 Module 4**	Men: *N* = 133	80.9 ± 7.7	0.0022	1.090 to 4.910
	Women: *N* = 102	77.9 ± 6.9		
**Year 1 Module 4 Anatomy**	Men: *N* = 133	76.7 ± 12	0.9514	
	Women: *N* = 102	76.6 ± 13		
**Year 1 Module 5**	Men: *N* = 133	81.9 ± 7.0	0.2878	
	Women: *N* = 102	80.9 ± 7.3		
**Year 1 Module 5 Anatomy**	Men: *N* = 133	69.2 ± 13	0.0634	
	Women: *N* = 102	65.9 ± 14		
**Year 1 Essentials of Clinical Medicine Fall Part 1**	Men: *N* = 133	82.2 ± 8.4	0.3901	
	Women: *N* = 102	83.1 ± 7.3		
**Year 1 Essentials of Clinical Medicine Fall Part 2**	Men: *N* = 133	79.8 ± 11	0.9417	
	Women: *N* = 102	79.9 ± 12		
**Year 1 Essentials of Clinical Medicine Spring Part 1**	Men: *N* = 133	79.5 ± 9.1	0.6	
	Women: *N* = 102	80.1 ± 8.1		
**Year 1 Essentials of Clinical Medicine Spring Part 2**	Men: *N* = 133	83.8 ± 10	0.5439	
	Women: *N* = 102	84.6 ± 9.9		
**Year 1 OCSE**	Men: *N* = 111	89.0 ± 4.3	0.3167	
	Women: *N* = 84	89.6 ± 3.9		
**Year 2 Module 1**	Men: *N* = 135	81.9 ± 6.1	0.0971	
	Women: *N* = 97	80.5 ± 6.6		
**Year 2 Module 2**	Men: *N* = 135	81.9 ± 6.5	0.452	
	Women: *N* = 97	81.2 ± 7.6		
**Year 2 Module 3**	Men: *N* = 135	81.7 ± 6.4	0.4791	
	Women: *N* = 97	81.1 ± 6.3		
**Year 2 Module 4**	Men: *N* = 135	82.2 ± 6.9	0.9108	
	Women: *N* = 97	82.3 ± 6.4		
**Year 2 Module 5**	Men: *N* = 135	82.5 ± 7.9	0.9275	
	Women: *N* = 97	82.6 ± 8.7		
**Year 2 Module 6**	Men: *N* = 135	84.3 ± 6.3	0.3954	
	Women: *N* = 97	85.0 ± 6.0		
**Year 2 Module 7**	Men: *N* = 114	82.9 ± 5.8	0.4337	
	Women: *N* = 77	82.2 ± 6.4		
**Year 2 OSCE**	Men: *N* = 114	90.9 ± 4.1	0.2131	
	Women: *N* = 77	91.6 ± 3.3		
**Year 2 Essentials of Clinical Medicine Fall Part 1**	Men: *N* = 135	79.6 ± 7.9	0.3605	
	Women: *N* = 97	78.6 ± 8.6		
**Year 2 Essentials of Clinical Medicine Fall Part 2**	Men: *N* = 135	78.9 ± 7.9	0.0987	
	Women: *N* = 97	77.1 ± 8.5		
**Year 2 Essentials of Clinical Medicine Spring Part 1**	Men: *N* = 135	82.0 ± 7.7	0.77	
	Women: *N* = 97	81.7 ± 7.7		
**Year 2 Essentials of Clinical Medicine Spring Part 2**	Men: *N* = 135	80.3 ± 7.3	0.2131	
	Women: *N* = 97	81.6 ± 8.5		
**USMLE Step 1**	Men: *N* = 130	235 ± 17	0.0122	1.32 to 10.68
	Women: *N* = 92	229 ± 18		
**Family Medicine**^a^	Men: *N* = 110	79.9 ± 6.3	0.1188	
	Women: *N* = 80	78.4 ± 6.8		
**Internal Medicine**^a^	Men: *N* = 113	79.4 ± 7.6	0.1580	
	Women: *N* = 80	77.8 ± 7.9		
**Neurology**^a^	Men: *N* = 108	82.0 ± 7.0	0.5662	
	Women: *N* = 77	81.4 ± 7.0		
**Obstetrics/Gynecology**^a^	Men: *N* = 105	80.5 ± 6.9	0.3591	
	Women: *N* = 76	81.4 ± 5.9		
**Pediatrics**^a^	Men: *N* = 110	80.5 ± 8.0	0.2881	
	Women: *N* = 79	79.3 ± 7.1		
**Psychiatry**^a^	Men: *N* = 110	82.6 ± 6.8	0.2171	
	Women: *N* = 80	83.8 ± 6.3		
**Surgery**^a^	Men: *N* = 112	78.7 ± 7.3	0.0232	0.331 to 4.469
	Women: *N* = 79	76.3 ± 6.9		
**Emergency Medicine**^a^	Men: *N* = 101	76.9 ± 7.4	0.9320	
	Women: *N* = 62	76.8 ± 7.0		
**Ambulatory**^a^	Men: *N* = 100	80.9 ± 6.2	0.0723	
	Women: *N* = 60	79.0 ± 6.8		
**USMLE Step 2 CK**	Men: *N* = 137	249 ± 15	0.3268	
	Women: *N* = 83	247 ± 14		

^a^All NBME subject exam scores reported are equated percent correct score

We analyzed summative assessment scores, including final exams, anatomy exams, and OSCEs, to determine if there was a difference in academic performance by gender in years 1 and 2 of our curriculum (pre-clerkship). We noted a consistent and significant difference (*p* = 0.004) in the scores on most module final exams in year 1, with men scoring higher than women. At the end of year 1 and throughout year 2, no difference in module final exams was noted between men and women. There was a difference for only one anatomy exam in an early module. No significant differences were found in performance on pre-clerkship OSCEs or Essentials of Clinical Medicine semester final exams (Table 2).

Analysis of Step 1 scores, which our students take at the end of the pre-clerkship curriculum (year 2), showed that there was a significant difference in performance, with men scoring higher (235 vs 229, *p* = 0.0122). In addition, we analyzed the equated percent correct scores for all NBME subject exams during clerkship curricula (years 3 and 4). Except for the surgery subject exam, there was no difference in performance by gender. Similar to what is observed in the national data [6], men did not perform better on the Step 2 CK (Table 2).

We identified gender gaps in academic performance at several key junctures at our campus: (1) pre-matriculation (science GPA and MCAT scores new format), (2) pre-clerkship internal exams (summative assessment scores in year 1), and (3) a national standardized exam (Step 1). These findings are consistent with those of other national and institutional studies [5, 6, 14].

Differences in performance by gender on internal assessments disappeared by the end of year 1 and were not present in year 2, yet reappeared on Step 1 scores (end of year 2), then disappeared again on the clerkship NBME subject exams (except in surgery) and Step 2 CK. These findings suggest that active learning at our campus did not contribute to the gender gap and may have closed the gap. Because women exhibit lower academic performance on Step 1 yet better performance on Step 2 CK, some have hypothesized a greater interest by women in clinical medicine and subject areas that have a larger representation on Step 2 CK than on exams that test mainly basic science knowledge, such as Step 1 [6]. However, the timeline of the gender gaps in performance from our cohorts of medical students does not support this theory. Given that Step 1 covers similar material to the internal assessments which equalize before the end of year 1, we predicted there would be no gender gap for our students. However, Step 1 scores from our students did exhibit a performance difference, favoring men, similar to national averages.

Historically, MCAT scores have played a role in medical school admission and, perhaps more critically, Step 1 scores have been used as a cutoff for consideration for residency programs. The 6-point difference in Step 1 scores favoring men nationally [5] and at our institution could result in less inclusion of women in the pool of candidates for residencies, especially the most selective specialties, which typically have lower representation of women. In 2022, Step 1 was changed to a pass/fail exam; however, a gender gap in scores could still exist and research on the effects of this change will be needed. If residencies substitute Step 2 CK scores in place of Step 1 in match considerations, it may result in less gender bias or may actually favor women.

Our future studies include expanding analysis of academic achievement by gender to all of the campuses of the Medical College of Georgia, since a limitation of this pilot study is that data from only one regional medical school campus was investigated. It will be interesting to determine whether there are regional and/or national trends in academic performance by gender. Qualitative studies that investigate the reasons for gender gaps in medical school performance will be needed to understand and then eliminate any existing differences in performance by gender. Only when we know where and why gender gaps exist can we begin to address equity issues in a meaningful way.

## Data Availability

All data generated or analysed during this study are included in this published article.
